# Diagnosis of acetaminophen and codeine poisoning in a dog using earwax analysis by headspace gas chromatography-mass spectrometry (HS/GC-MS)

**DOI:** 10.1007/s11259-026-11280-7

**Published:** 2026-05-23

**Authors:** Monica Chacon de Vicente, Alexandre Có Mangoni Barros, Gabriela Scarpin de Souza, Lais di Pauli Taborda Prado, João Marcos Gonçalves Barbosa, Rayanne Henrique Santana da Silva, Leidiane de Souza Gomes, Danieli Brolo Martins, Nelson Roberto Antoniosi Filho, Ana Flávia Machado Botelho

**Affiliations:** 1 School of Veterinary Medicine and Animal Science, Federal University of Goiás, Goiânia, Nova Veneza Highway, km 8, Samambaia Campus, Goiânia, 74690-900 Goiás Brazil; 2 Laboratory of Extraction and Separation Methods, Chemistry Institute, Federal University of Goiás (UFG), Campus II – Samambaia, Goiânia, 74690-900 GO Brazil

**Keywords:** Canine, Intoxication, Opioid, Paracetamol, Veterinary toxicology

## Abstract

Poisoning of companion animals resulting from exposure to analgesics intended for human use is a frequent and clinically relevant problem in veterinary practice, usually associated with administration without professional supervision. This can induce severe systemic toxicity. Diagnostic confirmation can be particularly challenging in cases presenting with nonspecific clinical signs or when conventional biological samples are unavailable. This case report describes the innovative use of earwax as a non-invasive biological matrix for toxicological confirmation in veterinary medicine. A 9-year-old male mixed-breed dog was admitted following a traumatic accident and subsequent administration by its owner of a human medication containing acetaminophen and codeine. Clinical evaluation revealed lethargy, hypersalivation, hyporexia, dehydration, and neurological abnormalities. Laboratory findings demonstrated neutrophilic leukocytosis and marked increases in hepatic enzyme activities, consistent with acute hepatocellular injury. Toxicological investigation using headspace gas chromatography–mass spectrometry detected acetaminophen-related signatures in an earwax sample, supporting suspected exposure to the drug. Intensive treatment was promptly initiated and included fluid therapy, antidotal treatment with N-acetylcysteine, opioid antagonism, analgesia, and supportive care. The patient showed progressive clinical improvement, with complete resolution of clinical signs and full recovery. This case highlights the significant risks of administering human medications to companion animals and underscores the need to improve owner awareness of self-medication practices. Furthermore, it demonstrates the diagnostic potential of earwax as a non-invasive biological matrix for toxicological confirmation, expanding the range of complementary diagnostic tools available for veterinary toxicology and supporting improved clinical decision-making in cases of suspected pharmaceutical intoxication.

## Background

Exposure to human medications is a leading cause of poisoning in small animals, commonly resulting from improper administration, off-label use, or accidental ingestion of inadequately stored pharmaceuticals. Anti-inflammatory drugs and analgesics are amongst the most frequently involved agents (Cortinovis et al. 2015). Acetaminophen (N-acetyl-p-aminophenol; paracetamol) is widely used for its analgesic and antipyretic effects (Chalasani et al. 2008) and represents a major cause of veterinary toxicological emergencies. Although cats are particularly susceptible, numerous cases have also been reported in dogs (Graham et al. 2013; MacNaughton 2003; McGill et al. 2013; Wallace et al. 2002).

Following oral administration, acetaminophen is rapidly absorbed, reaching peak plasma concentrations within approximately four hours (Ghanem et al. 2016). Hepatic metabolism occurs mainly via glucuronidation and sulfation, with renal excretion; however, a fraction is metabolized by cytochrome P450 to the reactive metabolite N-acetyl-para-benzoquinoneimine (NAPQI). Under normal conditions, NAPQI is detoxified by hepatic glutathione, but overdose leads to glutathione depletion, NAPQI accumulation, and severe hepatocellular injury (Aaronson 2000; Richardson 2000). Clinical signs include vomiting, apathy, facial edema, dyspnea, and, in severe cases, acute liver failure and death (Allen 2003; Dorigon et al. 2013). Early treatment is critical, with N-acetylcysteine (NAC) being the antidote of choice due to its role in restoring hepatic glutathione reserves (Thomer et al. 2019).

Codeine poisoning is less frequently reported, largely due to regulatory restrictions on its use in many countries. Codeine is an opioid analgesic for moderate pain and is primarily metabolized by cytochrome P450 2D6 into morphine, its main active metabolite (Kirchheiner et al. 2007; Nielsen and Van Hout 2015). Veterinary data on codeine poisoning are limited, but reported clinical signs of opioid overdose in dogs include miosis, respiratory depression, and mental depression (McMichael et al. 2022). Management relies on supportive care, assisted ventilation, and naloxone administration, with continuous monitoring in severe cases (Etherington et al. 2015).

Regarding the diagnosis of both poisonings, it is based on anamnesis, clinical findings, and laboratory analyses, with chromatographic techniques such as high-performance liquid chromatography, gas chromatography, and mass spectrometry used to confirm and quantify drug exposure (Richardson 2000; Boyer 2012; Thomer et al. 2019). In this study, we describe the clinical evolution, laboratory, ultrasonographic, and toxicological findings of an accidental poisoning of acetaminophen and codeine in a dog.

## Case presentation

A 9-year-old, 13.5-kg, male mixed-breed dog was admitted to the Veterinary Hospital of the Federal University of Goiás after being struck by a motorcycle and subsequently receiving an accidental overdose of paracetamol and codeine. According to the owner, the accident had occurred the previous day, with the main impact affecting the pelvic region. In an attempt to manage pain, the owner administered one tablet containing 500 mg of acetaminophen and an unknown concentration of codeine, resulting in an estimated acetaminophen dose approximately three times higher than the recommended therapeutic dose for dogs. Following administration, the dog developed prostration, mild hypersalivation, hyporexia, and reduced water intake. The animal had not urinated or defecated since the traumatic event.

On physical examination, the dog was prostrate, with hyperemic mucous membranes and a capillary refill time of 2 s. Rectal temperature was 38.7 °C, heart rate 108 beats/min, respiratory rate 28 breaths/min, and skin turgor was decreased. Exfoliative lesions involving the skin, subcutaneous tissue, and musculature (approximately 2 cm) were observed on the left flank and thigh and on the right hip region, accompanied by marked preputial edema.

Neurological evaluation revealed absent proprioception in the pelvic limbs, decreased patellar reflex in the right hind limb, reduced anal sphincter reflex, and absence of the cutaneous trunci reflex caudal to the iliac crest. Muscle tone and superficial pain perception in the pelvic limbs were reduced. Cranial nerve examination showed diminished pupillary and palpebral reflexes, without nystagmus or positional strabismus, as well as decreased sensitivity of the nasal planum and pinnae. Episodes of compulsive chewing and repetitive head movements were noted. Blood and cerumen samples were collected for laboratory analysis.

Laboratory tests revealed leukocytosis with segmented neutrophilia (total leukocytes: 21,900/mm³, reference: 6,000–17,000/mm³; segmented: 17,958/mm³, reference: 3,000–11,500/mm³) and an increase in band neutrophils (438/mm³, reference: 0-300/mm³), while myelocytes, metamyelocytes and basophils were absent (0–0/mm³), and eosinophils (219/mm³, 150-1,250/mm³), lymphocytes (2,628/mm³, 1,000–4,800/mm³) and monocytes (657/mm³, 150-1,350/mm³) were present. Total plasma protein was elevated at 8.4 g/dL (6.0–8.0 g/dL). The erythrogram showed an alteration only in the mean corpuscular hemoglobin concentration (MCHC) parameter of 31.9% (32.0–36.0%), while the other parameters remained normal, being red blood cell count of 7.66 tera/L (5.50–8.50 tera/L), hemoglobin of 16.4 g/dL (12.0–18.0 g/dL), hematocrit of 51.4% (37.0–55.0%), MCHC slightly below the reference value, with 31.9% (32.0–36.0%), mean corpuscular volume (MCV) of 67.1, red cell distribution width (RDW) of 14.3% (12.0–15.0%), and metarubricytes 1/100 leukocytes (0–1). Morphological observations included the presence of codocytes (++), slight hypochromia, neutrophils with cytoplasmic basophilia (+), Döhle bodies (+), and neutrophils with cytoplasmic vacuolization (rare). The platelet count was 464 giga/L (200–500 giga/L), mean platelet volume (MPV) of 6.8 fL (-), and platelet distribution width (PDW) of 15.8% (-).

Serum biochemistry showed an increase in alanine aminotransferase (ALT), with 702 IU/L (21–102 IU/L), alkaline phosphatase (ALP) of 530 IU/L (20–156 IU/L), and gamma-glutamyl transferase (GGT) of 7.0 IU/L (1.2–6.4 IU/L). Other parameters were also elevated, with total bilirubin at 0.89 mg/dL (0.10–0.50 mg/dL), direct bilirubin at 0.53 mg/dL (0.06–0.12 mg/dL), and indirect bilirubin at 0.36 mg/dL (0.01–0.49 mg/dL). Creatinine levels were normal at 0.94 mg/dL (0.50–1.50 mg/dL), and urea was 47 mg/dL (21.4–59.9 mg/dL). The reference values considered follow the ranges proposed by Harvey (2001) and Kaneko et al. (2008) (Table 1). X-rays of the pelvis and lumbosacral spine showed no significant bone alterations. Abdominal ultrasound revealed nephropathy in both kidneys and a possible hematoma or cyst in the right kidney.

**Table 1:** Blood count of a mixed-breed dog with suspected acetaminophen and codeine poisoning

Parameters	Results	Reference values
Erythrogram		
Red blood cells	7.66 tera/L	(5,50–8,50 tera/L)
Hemoglobin	16.4 g/dL	(12,0–18,0 g/dL)
Hematocrit	51.4%	(37,0 – 55,0%)
MCV	67,1 fL	(60,0–77,0 fL)
MCHC	31,9%	(32,0–36,0%)
RDW	14,3%	(12,0–15,0%)
Metarubricytes	1 /100 leuco	(0–1 leuco)
Leukogram		
Total leukocytes	21,900 /mm3	(6.000–17.000 /mm3)
Myelocytes	0 /mm3	(0–0 /mm3)
Metamyelocytes	0 /mm3	(0–0 /mm3)
Rods	438 /mm3	(0–300 /mm3)
Segmented	17.958 /mm3	(3.000–11.500 /mm3)
Eosinophils	219 /mm3	(150–1.250 /mm3)
Basophils	0 /mm3	(0–0 /mm3)
Lymphocytes	2.628 /mm3	(1.000–4.800 /mm3)
Monocytes	657 /mm3	(150–1.350 /mm3)
Platelet count		
Platelets	464 giga/L	(200–500 giga/L)
MPV	6.8 fL	—
PDW	15.8%	—
Serum biochemistry		
ALT	702 IU/L	21–102 IU/L
ALP	530 IU/L	20–156 IU/L
GGT	7.0 IU/L	1.2–6.4 IU/L
Creatinine	0.94 mg/dL	0.50–1.50 mg/dL
Urea	47 mg/dL	21.4–59.9 mg/dL
Total bilirubin	0.89 mg/dL	0.10–0.50 mg/dL
Direct bilirubin	0.53 mg/dL	0.06–0.12 mg/dL
Indirect bilirubin	0.36 mg/dL	0.01–0.49 mg/dL

*MCV,* mean corpuscular volume; *MCHC,* corpuscular hemoglobin concentration; *RDW*, red cell distribution width; *MPV,* mean platelet volume; *PDW,* platelet distribution width; *ALT,* alanine aminotransferase; *ALP,* alkaline phosphatase; *GGT,* gamma-glutamyl transferase. Reference values from Harvey 2001 and Kaneko et al. 2008

After cerumen collection, the sample was stored at − 20 °C until analysis by gas chromatography coupled with headspace injection mass spectrometry (HS/GC-MS). Then, the sample was weighed into 20 mL headspace vials for GC, which were sealed with hermetic rubber septum caps coated with polytetrafluoroethylene. As a quality control, a blank was analyzed after the addition of 3-methylcyclohexanone (Sigma-Aldrich^®^, St. Louis, MO, USA) as a chromatographic standard. The cerumen and quality control samples were then analyzed by HS/GC-MS.

The headspace analyzer was a Shimadzu AOC-5000 (Shimadzu^®^, Kyoto, Japan) equipped with a hermetic syringe with a volume of 2,500 µl and a VT32-20 tray (PAL System^®^, Zwingen, Switzerland), and an LHS0 Combi Pal preheating module with temperature and heating time control (PAL System^®^, Zwingen, Switzerland). The following parameters were used: (i) injection volume of 2,500 µl, (ii) injection rate of 1 ml s^− 1^, (iii) syringe temperature of 150 °C, (iv) preheating module agitation set at 500 rpm, (v) incubation temperature of 160 °C, (vi) agitation time of 60 min.

The GC used was a Shimadzu GCMS-QP2010 Ultra system (Shimadzu^®^, Kyoto, Japan), with the injector configured in splitless mode at 250 °C. The analysis of volatile compounds was performed using an NST-100 ms capillary column (25 m × 0.25 mm internal diameter × 0.3 μm film thickness; Nano Separation Technologies^®^, NST, São Paulo, Brazil) with a high polarity stationary phase (polyethylene glycol). The oven heating ramp program was defined as: 30 °C (5 min), then increased at 2 °C min^− 1^ to 40 °C (maintained for 5 min), again at 2 °C min^− 1^ to 50 °C (5 min), same heating gradient up to 120 °C; an increase of 6 °C min − 1 up to 200 °C (maintained for 5 min); and finally an increase of 5 °C min^− 1^ up to 250 °C, maintained for 10 min.

As a carrier gas, high-purity Helium gas (99.999%; 5.0, White Martins^®^, Goiânia, Brazil) was used with a linear velocity of 44.8 cm s^− 1^ and a flow rate of 1.36 ml min^− 1^. The MS operated in electron-impact ionization mode at 70 eV, with a scan speed of 1,600 us^− 1^ and a scan time of 0.3s. Putative annotation of volatile/semi-volatile compounds in cerumen was performed by comparing MS fragments with the NIST17 and NIST17s spectral libraries (National Institute of Standards and Technology^®^, Gaithersburg, MD, USA). For the suspect screening investigation, the sample was analyzed in two modes: (i) Scan mode with a scan of 40 to 500 *m/z*, (ii) Selected ion monitoring (SIM), monitoring the *m/z* ions 299, 229, 162 for codeine, the *m/z* ions 151, 109, 80, and 43 for acetaminophen, and *m/z* 112 and 69 for the chromatographic standard (3-methylcyclohexanone).

**Fig. 1:**
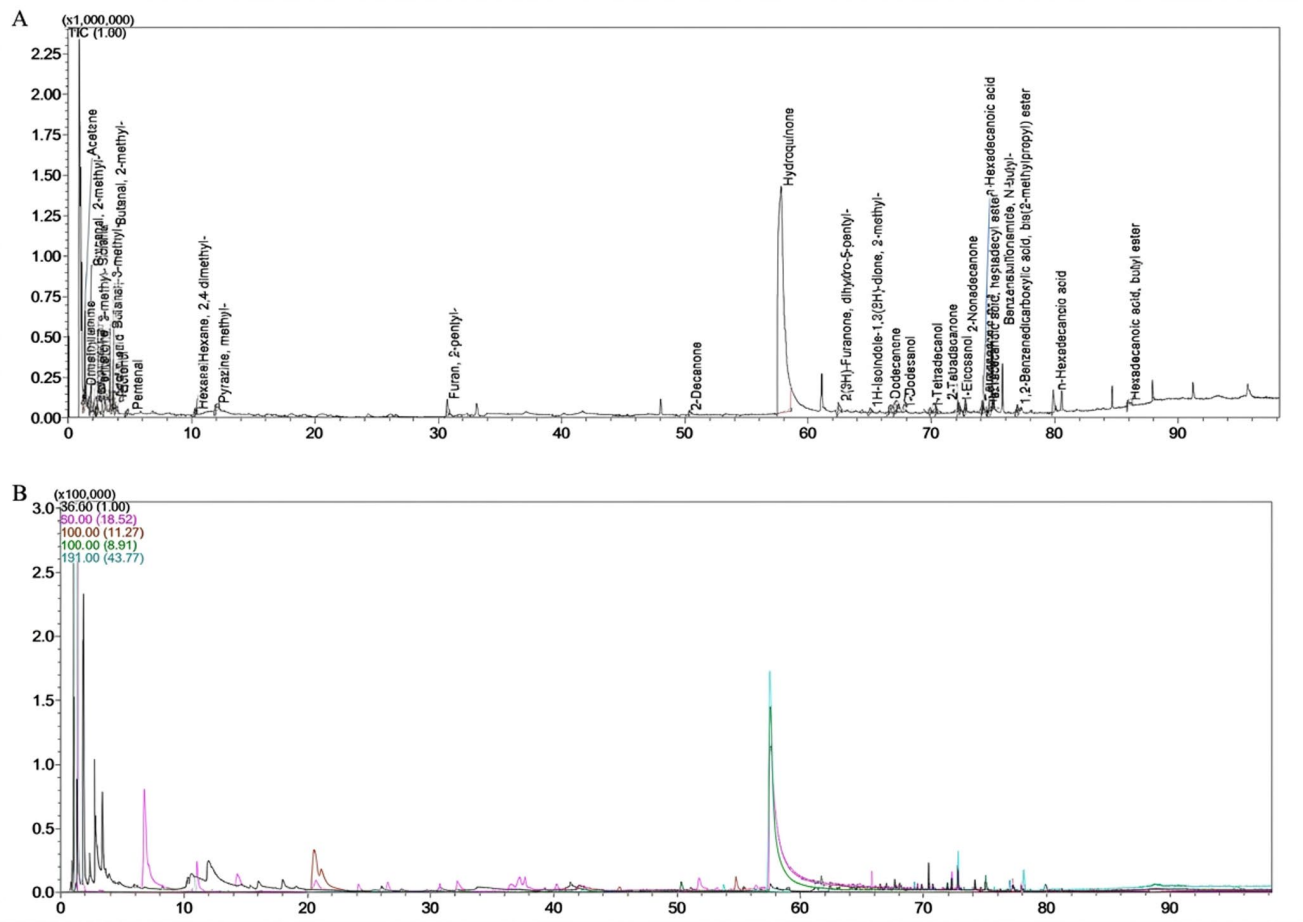
HS/GC-MS chromatographic profile of cerumen analysis from a 9-year-and-3-month-old mixed-breed dog, hit by a motorcycle and medicated with acetaminophen 37.03 mg/kg and codeine (dose not specified) the previous day, a potential source of drug poisoning. **A** HS/GC-MS Scan mode: 40–500 m/z, showing the hydroquinone peak at a retention time of 57.754 min. **B** Monitoring of characteristic paracetamol ions HS/GC-MS (SIM mode)

An acetaminophen (paracetamol)-related *m/z* fragments were observed in the cerumen sample analysis performed in SIM mode, co-eluting with hydroquinone, a metabolite and a thermal decomposition product of acetaminophen (de Freitas et al. 2021, 2025; Harrelson et al. 2012; Lara-Moreno et al. 2024; Qutob et al. 2022). No codeine-related fragments were detected using the HS/GC–MS method in this case. However, this finding should not be interpreted as evidence that codeine exposure did not occur. The non-detection might be due to limitations of the non-derivatized suspect-screening technique. Additionally, the pharmacokinetics, incorporation, and stability of codeine in canine cerumen are not well understood. As a result, the lack of detected codeine fragments does not rule out possible exposure, and the analytical findings should be interpreted together with the anamnesis, clinical signs, and conventional laboratory results (Fig. 1).

Treatment started immediately after hospitalization, with an intensive therapeutic approach. Fluid therapy was initiated with Ringer’s lactate at 3 mL/kg/hour, and dipyrone (0.67 mL, IV, QID) was also administered. To neutralize paracetamol toxicity, N-acetylcysteine was initially administered at 140 mg/kg, followed by 70 mg/kg every 6 h, diluted in saline and infused slowly. Analgesia was provided with meloxicam (0.1 mg/kg, IV, SID). Ondansetron (1 mg/kg, IV, BID) was given, but due to the persistence of the emetic condition, maropitant (1 mg/kg, SC, SID) was administered. Naloxone was also included at a dose of 0.067 mL intravenously once a day to reverse the possible adverse effects of opioids. Skin wounds were treated with a mixed ointment containing Gentamicin 0,5 g; Sulfanilamide 5,0 g; Sulfadiazine 5,0 g; Urea 5,0 g; Vitamin A 12,000 UI, applied topically twice a day (BID) after cleansing with saline solution. Blood glucose levels were checked three times a day, as was food intake.

After 28 h of intensive treatment, the animal exhibited clinical improvement, characterized by reduced lethargy and salivation, as well as normalization of neurological function. The patient was discharged with instructions to continue treatment at home, which included: metamizole (25 mg/kg, PO, 3 times a day), meloxicam (1.5 mg, PO, once a day), tramadol (50 mg, PO, once a day), gabapentin (300 mg, PO, once a day), cyproheptadine and vitamins B and C complexes (1.3 mL, PO, once a day), ondansetron (8 mg, PO, once a day), and amoxicillin and potassium clavulanate (200 mg and 50 mg, respectively, PO, once a day). Topical application of gentamicin 0.5 g, sulfanilamide 5.0 g, sulfadiazine 5.0 g, and vitamin A 12,000 IU was maintained twice daily. On the fifth day after discharge, the owner returned for reassessment and was instructed to continue oral and topical antimicrobial therapy for an additional 10 days. No apparent adverse outcomes were observed.

### Discussion and conclusions

This report describes a case of paracetamol and codeine poisoning in a dog resulting from improper administration by the owner. The main clinical findings included prostration, mucous membrane hyperemia, and neurological abnormalities, while laboratory evaluation revealed leukocytosis with segmented neutrophilia and increased liver enzyme activities, with no evidence of renal impairment. Confirmatory poisoning detection was achieved using an innovative HS/GC–MS as a complementary approach, employing earwax as the biological matrix.

Poisoning by analgesics is common in dogs, especially due to self-administration of drugs intended for human use without veterinary supervision (Cortinovis et al. 2015). Fixed-dose combinations of codeine and acetaminophen are widely prescribed in human medicine (KuKanich 2016), but remain restricted and rarely documented in veterinary practice. The dog in the present case had unspecified clinical signs, such as prostration, hypersalivation, hyporexia, and hyperemia. Dehydration was confirmed by both skin turgor and serum protein levels. Several neurological signs were also observed, mainly associated with proprioception and reflexes, including compulsive chewing and repetitive head movements.

The main laboratory findings included leukocytosis driven by neutrophilia, accompanied by morphological changes such as basophilia, cytoplasmic vacuolization, and Döhle bodies, consistent with an acute inflammatory response likely secondary to extensive skin lesions. Serum biochemistry revealed marked increases in alkaline phosphatase (ALP) and alanine aminotransferase (ALT), along with a mild increase in gamma-glutamyl transferase (GGT), indicative of hepatocellular and biliary injury. These alterations were corroborated by elevated bilirubin concentrations. Although abdominal ultrasonography did not reveal significant abnormalities, the overall clinicopathological findings support a diagnosis of acute liver injury.

Hepatic damage has been documented in experimental models of acetaminophen overdose, where high doses induce fulminant hepatitis and correlate with increased serum bilirubin and ALT activity, as well as histopathological evidence of hepatic necrosis. The mechanism underlying this hepatotoxicity involves hepatic metabolism of acetaminophen via cytochrome P450 enzymes to the reactive metabolite NAPQI, which, when present in excess, depletes glutathione and binds covalently to cellular proteins, leading to oxidative stress and hepatocyte death (Chidiac et al. 2023).

The patient’s response to treatment was considered satisfactory, with progressive improvement in clinical signs. The reduction in prostration and the return of proprioception in the pelvic limbs indicate an improvement in the neurological condition, possibly related to drug metabolism and excretion, as well as the resolution of possible tissue hypoxia. The timely administration of NAC (Chidiac et al. 2023) and naloxone (Shaw et al. 2019), combined with fluid therapy, adequate analgesia, and supportive measures (DeClementi 2018), proved fundamental in preventing irreversible injuries and promoting the patient’s clinical recovery, demonstrating the effectiveness of the therapeutic protocol adopted.

To confirm the diagnosis, complementary analytical techniques are often required. Acetaminophen exposure has previously been confirmed in human biological fluids using gas chromatography–mass spectrometry (GC–MS) (Biscevic-Tokic et al. 2015), while codeine has been detected in canine plasma by liquid chromatography–tandem mass spectrometry (LC–MS/MS) (Hu et al. 2011). To the best of our knowledge, this is the first report describing the use of a non-invasive earwax (cerumen) sample for the detection of these analgesics in veterinary practice. In recent years, earwax has emerged as an alternative biological matrix for toxicological diagnosis in both humans (Shokry et al. 2017) and animals (Barbosa et al. 2021), either through direct detection of xenobiotics or via identification of relevant metabolites using metabolomic approaches.

Although no codeine-derived metabolites were detected, earwax proved to be a viable non-invasive sample for acetaminophen and its metabolite, hydroquinone, a thermal degradation product of acetaminophen. The detection of paracetamol in cerumen by HS/GC–MS supports the value of complementary diagnostic methods in cases of suspected intoxication, particularly when anamnesis is inconclusive or multiple substances are involved. Suspect screening HS/GC–MS analysis indicated paracetamol-related *m/z* signatures in the earwax sample, supporting exposure to the drug in the clinical context. Acetaminophen was detected in selected ion monitoring (SIM) mode; however, co-elution was observed with a peak corresponding to hydroquinone (retention time: 57.754 min), a metabolite and a thermal degradation product of acetaminophen, which was detected with greater intensity in this sample and showed similarity greater than 95%.

Although the conversion of NAPQI to hydroquinone is considered a secondary and not fully established metabolic pathway, this transformation is chemically plausible. Dahlin et al. (1984) reported the formation of p-benzoquinone as a secondary hydrolysis product of NAPQI, and hydroquinone-like structures generated during the redox cycling of acetaminophen derivatives have been described in electrochemical studies (de Freitas et al. 2021, 2025; Harrelson et al. 2012; Lara-Moreno et al. 2024; Qutob et al. 2022; Shayani-Jam and Nematollahi 2010). These findings, associated with the potential thermal degradation linked to headspace extraction, might support the interpretation of the observed chromatographic profile and reinforce the relevance of HS/GC–MS as a complementary tool in veterinary toxicological diagnosis.

This case highlights important clinical implications for veterinary practice by highlighting the risks associated with the inappropriate administration of human medications to animals. The unsupervised use of paracetamol and codeine exposed the dog to a toxic dose of paracetamol and the potential adverse effects of codeine, reflecting a common lack of owner awareness regarding the risks of self-medicating pets. In addition, this report demonstrates the novelty and diagnostic value of using earwax as a non-invasive biological matrix for toxicological confirmation. This complementary diagnostic tool should be considered when standard sample collection is limited or unfeasible, such as in cases of anuria or restricted availability of biological material, rather than replacing established diagnostic protocols.

## Data Availability

All relevant data are within this paper. The datasets generated during the current case study are available from the corresponding author on reasonable request.

## Abbreviations

ALP: Alkaline phosphatase
ALT: Alanine aminotransferase
BID: Twice daily
GC: Gas chromatography
GC–MS: Gas chromatography–mass spectrometry
GGT: Gamma-glutamyl transferase
HS/GC–MS: Headspace gas chromatography–mass spectrometry
IV: Intravenous
LC–MS/MS: Liquid chromatography–tandem mass spectrometry
MCHC: Mean corpuscular hemoglobin concentration
MCV: Mean corpuscular volume
MPV: Mean platelet volume
MS: Mass spectrometry
NAC: N-acetylcysteine
NAPQI: N-acetyl-para-benzoquinone imine
PDW: Platelet distribution width
PO: Oral administration
QID: Four times daily
RDW: Red cell distribution width
SC: Subcutaneous
SID: Once daily
SIM: Selected ion monitoring
UI: International units
